# Delayed modulation of alpha band activity increases response inhibition deficits in adolescents with AD(H)D

**DOI:** 10.1016/j.nicl.2024.103677

**Published:** 2024-09-30

**Authors:** Katharina Graf, Roula Jamous, Moritz Mückschel, Annet Bluschke, Christian Beste

**Affiliations:** a Cognitive Neurophysiology, Department of Child and Adolescent Psychiatry, Faculty of Medicine, TU, Dresden, Germany

**Keywords:** ADHD, Response inhibition, EEG, Theta band activity, Alpha band activity, MVPA

## Abstract

Deficiencies in inhibitory control are one of the hallmarks of attention-deficit-(hyperactivity) disorder (AD(H)D). Response inhibition demands can become increased through additional conflicts, namely when already integrated representations of perception–action associations have to be updated. Yet, the neural mechanisms of how such conflicts worsen response inhibition in AD(H)D are unknown, but, if identified, could help to better understand the complex nature of AD(H)D-associated impulsivity. We investigated both behavioral performance and EEG activity in the theta and alpha band of adolescents (10–18 years of age) with AD(H)D (n = 28) compared to neurotypical (NT) controls (n = 33) in a conflict-modulated Go/Nogo paradigm. We used multivariate pattern analysis (MVPA) and EEG-beamforming to examine how changes in representational content are coded by oscillatory activity and to delineate the cortical structures involved in it. The presented behavioral and neurophysiological data show that adolescents with AD(H)D are more strongly affected by increased response inhibition demands through additional conflicts than NT controls. Precisely, AD(H)D participants showed higher false alarm rates than NT controls in both, non-overlapping and overlapping Nogo trials, but performed even worse in the latter. This is likely due to an inefficient updating of representations related to delayed modulations of alpha band activity in the ventral stream and orbitofrontal regions. Theta band activity is also modulated by conflict but was not differentially affected in the two groups. By this, the present study provides novel insights into underlying neurophysiological mechanisms of the complex nature of response inhibition deficits in adolescents with AD(H)D, stressing the importance to examine the interplay of theta and alpha band activity more closely to better understand inhibitory control deficits in AD(H)D.

## Highlights

• New neurophysiological insights into AD(H)D’s inhibitory control deficits.
• AD(H)D adolescents struggle more with response inhibition under high cognitive load.
• Unsuccessful inhibition is linked to inefficient updating of mental representations.
• Delayed alpha band modulation, not changes in theta band, underlies these deficits.

## 1 Introduction

Attention-deficit(−hyperactivity) disorder (AD(H)D) is one of the most prevalent psychiatric disorders of childhood and adolescence and is characterized by three core symptoms (inattention, hyperactivity, and impulsivity) (American Psychiatric Association, 2013, Faraone et al., 2015, Polanczyk et al., 2007). Behavioral and neurophysiological evidence suggests that AD(H)D is associated with deficiencies in cognitive and specifically inhibitory control (Ahmadi et al., 2014, Albrecht et al., 2014, Bluschke et al., 2018, Hart et al., 2013, Huizenga et al., 2009, Johnstone et al., 2009, Lijffijt et al., 2005, McAuley et al., 2014, Rubia et al., 2005, Smith et al., 2004, van Rooij et al., 2015, Willcutt et al., 2005). Response inhibition processes refer to the ability to suppress irrelevant information while prioritizing relevant information. Thus, it is necessary for goal-directed action in daily life and for suppressing inappropriate behavior (Bari and Robbins, 2013, Gratton et al., 2018). Abnormalities in these processes have even been suggested to be one of the core neurocognitive deficits of AD(H)D that further contribute to main (hyperactivity, impulsivity, attention regulation) but also to more secondary deficits of the disorder (e.g., working memory or self-regulatory deficits; Barkley, 1997, Nigg, 2001). While research shows that inhibitory control deficits are prevalent in AD(H)D (e.g., Aguiar et al., 2010, Alderson et al., 2007), more recent studies suggest that these are neither central nor specific to AD(H)D (Barkley, 2014, Hoegl et al., 2012, Pievsky and McGrath, 2018, Wright et al., 2015). For example, other psychiatric disorders (e.g., autism spectrum disorder) can be affected too, and not all individuals diagnosed with AD(H)D suffer from these deficits (Barkley, 2014, Pievsky and McGrath, 2018). A great deal of research has been done on the neurophysiological processes underlying inhibitory control deficits in AD(H)D (e.g., Albrecht et al., 2014, Hoegl et al., 2012, Johnstone et al., 2013, Johnstone et al., 2009, Johnstone and Clarke, 2009, Vahid et al., 2019). Studying in which contexts these deficits in AD(H)D might arise and become even greater or less pronounced has however been left out so far. Identifying neural mechanisms affecting these processes may help patients with AD(H)D to handle their deficits more efficiently and to create environments that attenuate the occurrence of these deficits.

One relevant process may be the increase of response inhibition demands through additional conflicts that lead to a decline in performance even in neurotypical (NT) individuals (Chmielewski et al., 2016, Prochnow et al., 2022b, 2022a, 2021). Generally, dealing with conflicts increases the necessity to update existing representations of perceptions and their associated actions (Colzato et al., 2006, Frings et al., 2020, Hommel, 2009, Hommel, 2004). Such updating processes incorporate the binding and retrieval of integrated representations of perception–action associations (Frings et al., 2024, Frings et al., 2020, Hommel et al., 2001). Inhibitory control and processes managing integrated perception–action associations share neurophysiological systems and functional anatomical structures (Chmielewski and Beste, 2019). Particularly, oscillatory activity in the theta (∼4–7 Hz) and alpha (∼8–12 Hz) frequency bands is modulated by changes in demands on inhibitory mechanisms (Bastiaansen and Hagoort, 2003, Klimesch, 1999). Moreover, increased frontal theta band activity (TBA) is associated with better performance in conflict monitoring (Cavanagh et al., 2009, Eschmann et al., 2018, Trujillo and Allen, 2007) as well as response inhibition (Funderud and I., Lindgren, M., Løvstad, M., Endestad, T., Voytek, B., Knight, R.T., Solbakk, A.-K. , 2012, Kamarajan et al., 2004, Nigbur et al., 2011). On the other hand, alpha band activity (ABA) likely implements control by suppressing task-irrelevant information in working memory, facilitating the processing of relevant information (Bonnefond and Jensen, 2012, Foxe and Snyder, 2011, Janssens et al., 2018, Klimesch, 2012, Klimesch et al., 2007).

Notably, recent findings suggest that the management of integrated perception–action representations depends on a fine-tuned interplay of TBA and ABA (Beste et al., 2023). Thus, this interplay might also be important for successful response inhibition in cases where it seems necessary to modify integrated perception–action representations (Prochnow et al., 2022b, Prochnow et al., 2022a, Prochnow et al., 2021, Wendiggensen et al., 2023): TBA appears to be involved in storing the internal representations into an episodic memory trace as well as in retrieving and updating this entry. In turn, ABA seems to function as a dynamic top-down control over TBA during response inhibition (Wendiggensen et al., 2023). Along these lines, increased TBA and decreased ABA are evident when more cognitive control and increased monitoring of cognitive processes is necessary (Beste et al., 2023, Chmielewski and Beste, 2019, Prochnow et al., 2022b, Prochnow et al., 2022a, Prochnow et al., 2021, Wendiggensen et al., 2023). Conversely, when cognitive load is low (i.e. when no updating of perception–action representations is necessary), decreased TBA and increased ABA likely allow a more efficient processing of information (Beste et al., 2023, Chmielewski and Beste, 2019, Klimesch, 2012, Klimesch et al., 2007, Prochnow et al., 2022a, 2021). Therefore, a closer consideration of the contribution of TBA and ABA (and their interplay) to the formation and updating of perception–action representations is crucial. This is a necessary step towards a better understanding of the inhibitory control deficits in AD(H)D and their underlying neural mechanisms.

In AD(H)D, previous findings demonstrate a significant contribution of TBA to executive functioning deficits (McLoughlin et al., 2022). Several lines of evidence suggest a weakened mid-frontal TBA in tasks with high conflict processing demands (Johnson et al., 2008, Lenartowicz et al., 2014, McLoughlin et al., 2014) and inhibitory control (Pertermann et al., 2019, Yordanova et al., 2013) in AD(H)D. Similarly, dysfunctions in ABA have also been connected to failed suppression of irrelevant information (Ippolito et al., 2022, Lenartowicz et al., 2019, 2014). However, an integrated mechanistic understanding of the relevance of the interplay of TBA and ABA for inhibitory control deficits in AD(H)D is missing.

In the current study, we therefore ask whether and how response inhibition deficits in AD(H)D are modulated by different degrees of conflict. We investigate how both the behavioral performance and the oscillatory activity of adolescents with AD(H)D differ from NT controls regarding their performance on a well-established conflict-modulated Go/Nogo paradigm (Chmielewski and Beste, 2019). The task includes varying conditions of stimulus feature overlap between Go and Nogo trials (Chmielewski and Beste, 2019). In this task, letters and words in various font colors served as stimuli. Less cognitively demanding conditions included Go and Nogo trials which did not overlap in stimuli and had entirely different wording and font colors (non-overlapping trials). Conversely, high cognitive load conditions included Go and Nogo trials in which the font colors or words overlapped (overlapping trials). Response inhibition is compromised when a Nogo trial has been previously associated with and shares visual features with a Go trial. By that, the task combines the possibility of examining cognitive conflict and inhibitory control processes simultaneously (Prochnow et al., 2022b, 2022a, 2021). Inhibitory control processes are indicated by the percentage of errors (i.e., false alarm rate) in the Nogo conditions (independent of the stimulus feature overlap between Go and Nogo trials). Cognitive conflict processes on the other hand are indicated by the false alarm rate in the non-overlapping compared to the overlapping trials. The overlapping trials present the cognitively more demanding trials (independent of Go and Nogo trials). The difference of non-overlapping minus overlapping conditions is referred to as the conflict effect.

We hypothesize that the already impaired response inhibition processes in AD(H)D will be particularly dysfunctional when a high degree of conflict is added. On the neurophysiological level, we assume that AD(H)D patients are less efficient at up/downregulating their TBA and ABA depending on the different demands posed onto them during the different conditions. This would suggest a deficient ability to monitor cognitive processes and an inadequate updating of mental representations of working memory content compared to NT adolescents. Furthermore, we assume for AD(H)D adolescents to show smaller ABA modulations in response to the less demanding conditions. By this, reflecting a deficient ability to inhibit task-irrelevant information.

We use time–frequency analysis to investigate the modulation of ABA and TBA. As previously stated, these frequency bands have been found to be sensitive to changes in task demands, especially to manipulations of cognitive load and inhibitory mechanisms (Bastiaansen and Hagoort, 2003, Klimesch, 1999). We further used multivariate pattern analysis (MVPA) to study the temporal stability of the oscillatory activity by decoding the representational differences during different levels of cognitive load across the whole trial. By that, we are able to capture more complex and precise neurophysiological pattern differences (Carlson et al., 2019, Fahrenfort et al., 2018). More specifically, temporal generalization MVPA enables us to examine when and for how long the mental representations are significantly distinguishable in oscillatory activity (Fahrenfort et al., 2018, King and Dehaene, 2014, Takacs et al., 2021). Thus, this provides us with information about the stability of these representations over time. MVPA will also be used to investigate the interplay between TBA and ABA: by using one frequency band as training data to predict the other frequency band to examine if and when there are significant similarities between the two. Additionally, we perform source-localization analysis (DICS beamforming analysis) to localize brain regions associated with activity modulations for the contrast between non-overlapping and overlapping Nogo trials in both groups (Gross et al., 2001, Westner et al., 2022). By that, we want to further explore whether possible group differences in time–frequency data might relate to potential differences in activated brain structures. Beamforming is a widely accepted analytical approach in the field of EEG data processing for source localization (Dippel et al., 2016, Pscherer et al., 2019, Westner et al., 2022).

## 2 Materials and methods

To ensure comparability of the results, analyses were conducted in accordance with the methods applied by Wendiggensen et al. (2023) and Prochnow et al. (2024).

### 2.1 Sample

N = 28 adolescents (10–18 years) with confirmed AD(H)D diagnoses (AD(H)D group) according to the ICD-10 were included in the study. In addition, a second group of N = 33 neurotypical controls (NT group) was included. In both groups, the mean IQ was in the average range. Please see Table 1 for full demographic information.

**Table 1:** Demographics of the Sample (N_NT_ = 33, N_ADHD_ = 28).

Demographic variables	NT Group (m ± SE)	ADHD Group (m ± SE)	Significance test
Gender (Valid N)			*χ^2^*(1) = 6.57, *p* = 0.010*
Male	14	21	
Female	19	7	
Age	13.97 ± 0.39	13.32 ± 0.23	*U* = 377.00, *Z =* − 1.25*, p = 0*.212
IQ	110.81 (± 2.08)	104.39 (± 2.89)	*t*(58) = 1.833, *p* = 0.072
Regular medication use (Valid N)	0	12	
Medication use on the day of testing (Valid N)	0	3	
Reported AD(H)D diagnoses (Valid N)			
ADHD	0	18	
ADD	0	9	
Hyperkinetic conduct disorder	0	1	
Other reported psychiatric diagnoses (Valid N)			
Enuresis Nocturna	2	0	
Dyslexia	0	2	
Dyscalculia	0	2	
Developmental coordination disorder	0	2	
Bipolar disorder	0	1	
Conners’ Rating Scale − Parent			
Inattention	49.13 ± 1.05	67.42 ± 1.33	*U* = 30.50, *Z* = − 6.06, *p* < 0.001***
Hyperactivity/Impulsivity	48.59 ± 1.10	64.19 ± 1.43	*U* = 54.50, *Z* = − 5.66, *p* < 0.001***
Learning Problems	45.34 ± 1.42	58.42 ± 2.08	*U* = 140.00, *Z* = − 4.32, *p* < 0.001***
Executive Functioning	47.47 ± 1.08	66.54 ± 1.29	*U* = 23.00, *Z* = − 6.16, *p* < 0.001***
Aggression	52.22 ± 1.14	60.69 ± 1.80	*U* = 196.50, *Z* = − 3.46, *p* < 0.001***
Peer Relations	52.22 ± 1.14	62.81 ± 1.57	*U* = 120.00, *Z* = − 4.66, *p* < 0.001***

Note. *.01 ≤ p < 0.05; ** 0.001 ≤ p < 0.01; *** p < 0.001.

Abbreviations: NT = neurotypical, AD(H)D = attention-deficit(−hyperactivity)-disorder, SE = standard error.

All participants were recruited by means of an in-house database, or by advertisement. With the permission of the (potential) participants and their legal guardians, contact and medical information (e.g., psychiatric diagnoses) of these participants are kept in this database. Patients in this database were recruited from the outpatient clinic associated with our department. (Potential) participants responding to the advertisement were asked to provide contact information and any information that would be required to determine their eligibility for the study, including psychiatric illnesses. Interest in participation as well as inclusion/exclusion criteria in the current study was asked from all (potential) participants and their legal guardians via a short telephone interview. To further have a more precise overview of the presence or absence of symptoms and potential comorbidities, parents were asked to complete online questionnaires, particularly concerning AD(H)D symptoms.

Patients with AD(H)D who were taking medication for AD(H)D were only eligible to participate if they took only one dose of the active substance methylphenidate in the morning. To minimize or even eliminate the effects of the medication, appointments of the study were scheduled for the late afternoon (Morton and Stockton, 2000). Parent’s report states that of N = 12 AD(H)D patients taking regular medication with methylphenidate, only N = 3 of those reported to have taken medication on the day of testing.

Of originally 41 NT participants who reported to not have any prior psychiatric or neurological diagnoses or related previous treatment, seven participants were excluded before data analysis due to abnormally high scores in the AD(H)D questionnaires. One further participant was excluded due to being a strong outlier in all Go trials. Of originally 33 AD(H)D participants, five participants were excluded prior to data analysis due to several reasons: two participants withdrew study participation, two participants had too poor EEG data quality, and one participant was excluded as an outlier during behavioral analysis. Power calculations with the G*Power software package revealed that a minimum of N = 54 participants (N = 27 per group) is required to obtain a minimum effect size of f = 0.25 (this equals η_p_^2^ = 0.06) with a power of 95 % for studying the interaction ‘feature overlap x group’ (Faul et al., 2007). Hence, the sample size at hand should be reasonably powered to observe the desired effects. The study was approved by the ethics committee of the Faculty of Medicine of TU Dresden.

### 2.2 Task

Similar to Wendiggensen et al. (2023), we used an established conflict Go/Nogo task (Chmielewski and Beste, 2019, Petruo et al., 2019, Prochnow et al., 2022a, Prochnow et al., 2021, Wendiggensen et al., 2023) which combines a classical Go/NoGo task with overlapping and non-overlapping conditions, thereby adding a conflict element. Stimuli consist of letters displayed in different font colors, with Go and NoGo trials sharing one (i.e., overlapping condition) or none of those letter/color features (i.e., non-overlapping condition). Thus, trials with overlapping features were defined by the characteristics of the stimuli (i.e., letters or color) being applied for both Go and Nogo trials.

For each Go and Nogo trial, there were two different kinds of overlap/conditions. Stimuli displaying the word “DRÜCK” (German for “press”), could be both: A Go signal (white font) or a Nogo signal (blue font). Similarly, stimuli displaying the letters “XXXXX” could be both: A Go signal (blue font) or a Nogo signal (white font). This way, those stimuli were ambiguous regarding the necessary reaction, namely whether a response is required (Go signal) or whether a response needs to be inhibited (Nogo signal). Thus, those stimuli call for an update of the internal representations of the affiliated features due to the re-coupling of stimulus characteristics and the corresponding response. By that, false alarm rates are expected to increase in the overlapping Nogo trials. On the other hand, the word “PRESS” in green font color was exclusively a non-overlapping Go signal, while the word “STOPP” in red font color was exclusively a non-overlapping Nogo signal. Consequently, those stimuli were unambiguous and therefore required a lower cognitive load.

**Fig. 1:**
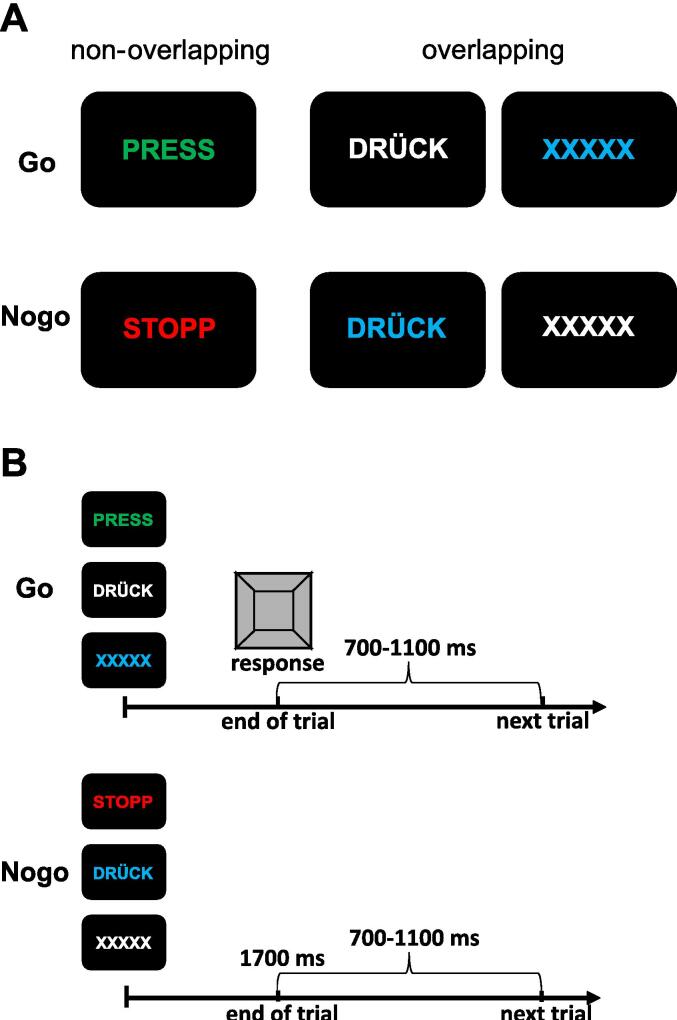
Schematic illustration of the conflict Go/Nogo paradigm. The exact stimuli of the task are shown in Fig. 1A, while the structure and timing are displayed in Fig. 1B.

Participants performed 30 practice trials. Stimuli were presented for 450 ms and trials ended after 1700 ms, or until a response had been given by the participant. The inter-trial interval was jittered between 700 and 1100 ms. Between trials, a white fixation cross was shown in the center of the screen. Participants were instructed to press the space key for Go stimuli, while not pressing any key for NoGo stimuli. Go and NoGo trials were pseudo-randomly displayed in a ratio of 70:30 to generate a prepotent responding tendency. Both Go conditions consisted of 184 trials, whereas both NoGo conditions consisted of 76 trials. The exact stimuli are shown in Fig. 1A, while the structure and timing are displayed in Fig. 1B. For analysis purposes, both overlapping conditions were combined for Go and Nogo trials.

### 2.3 EEG recording and preprocessing

EEG data (recorded using 60 Ag/AgCl electrodes, ground at θ = 58, φ = 78, reference at Fpz during recording, while it was changed to average reference at the beginning of preprocessing, impedance < 5 kΩ) was stimulus-locked (−2500 to 3000 ms) and segmented into non-overlapping and overlapping correct Nogo trials (i.e., trials in which inhibition was successful) using BrainVision Analyzer 2.1. Beforehand, after changing the sample rate to 256 Hz and applying a band-pass filter (0.5 – 40 Hz), technical artifacts were eliminated based on visual inspection during manual preprocessing, while recurring artifacts (e.g., pulse artifacts and horizontal and vertical eye movements) were removed by an independent component analysis (ICA, infomax algorithm). After baseline correction (−200 to 0 ms), an automated artifact rejection excluded any remaining artifacts (i.e., less than 0.5 μV within a 100 ms period or amplitudes greater than ± 150 µV) and designated them as bad intervals 200 ms before and 200 ms after the event. Segments including these bad intervals were rejected for data analyses. For all following analyses, segments of 0 to 1000 ms after stimulus onset were used. The cut-off was set at a minimum of 20 segments left for analysis.

### 2.4 Time-frequency decomposition

For comparing oscillatory activity in the different Nogo conditions within and between both groups, a time–frequency transformation with Morlet wavelets (Morlet parameter of 5) was performed. In steps of 1 Hz, the analysis was performed in the range of 1 to 30 Hz. To determine the electrodes and time windows with significant differences in TBA (averaged over 4–7 Hz) and ABA (averaged over 8–12 Hz) between the two conditions of Nogo trials and between the two groups, non-parametric cluster-based permutation tests were performed as implemented in FieldTrip (Maris and Oostenveld, 2007). To define a cluster, two consecutive time points or two adjacent EEG channels were needed. Using the Monte Carlo method, 1000 random draws were made to approximate the reference distribution of the permutation test. If the corresponding *p* < 0.05, a cluster was regarded as significant.

#### 2.4.1 MVPA

We aimed at examining how the classification performance into a non-overlapping vs. overlapping trial based on the oscillatory activity differs between the two groups. Therefore, three different classifications were investigated within each group: the classifier being 1) trained and tested on the time course of TBA; 2) trained and tested on the time course of ABA; 3) trained on the time course of TBA and tested on the time course of ABA. The first two classifications allow us to investigate at what time non-overlapping and overlapping trials can be clearly distinguished. Further, the third classification enables us to examine the interplay of the two frequency bands.

For each of the investigated approaches, we used the MVPA-light toolbox (Treder, 2020) and trained a binary classifier to discriminate between the overlapping and non-overlapping conditions. In general, we adopted the default settings of the toolbox except for choosing a two-class L1-Support Vector Machine (SVM) classifier. We decided to apply the SVM classifier because it is more robust to outliers and therefore better suited to noisy and non-Gaussian data than the LDA classifier (Treder, 2020). Furthermore, we used a cross-validation method with five folds. The MVPA analyses were performed on each participant’s dataset with the time course in each electrode as a feature vector (i.e., as input). First, we performed an MVPA to investigate at which time points after stimulus presentation the classifier can discriminate between both conditions based on the pattern of the source-reconstructed time–frequency data (i.e., theta power and alpha power, respectively) across all electrodes. The results were displayed via the area under the ROC curve (AUC). Additionally, a temporal generalization analysis was carried out with the same feature vector to obtain information on the temporal dynamics of the activation patterns. We performed cluster-based permutation tests (based on Wilcoxon tests with a threshold set at *p* = 0.05) to test at which time points the AUC differed significantly from the chance level of 50 % correct classification. To approximate the reference distribution, 1000 random draws were used. The statistical values of the cluster-based permutation tests were computed as the sum of all Wilcoxon-values within the time points.

### 2.5 Beamforming analysis (DICS)

To assess the source activity underlying TBA and ABA for the contrast between non-overlapping and overlapping Nogo trials in both groups, Dynamic Imaging of Coherent Sources (DICS) beamforming (Gross et al., 2001) was conducted to localize brain regions associated with activity modulations in the different frequency bands. This was done to explore whether potential differences in frequency band activations might arise from differences in activated brain structures. Common spatial filters within each group were computed from the cross-frequency spectra of a Fast Fourier Transformation (FFT) on the averaged theta (4–7 Hz) and alpha (8–12 Hz) frequency bands to compute the DICS beamforming. The DICS beamformer mapped the frequency band activity onto a grid with an even spacing of 0.5 cm based on the FieldTrip toolbox's forward model template that is based on the standard Montreal Neurological Institute (MNI) space. For source reconstruction of single conditions, the Neural Activity Index (NAI) was then computed to remove the center-of-the-head bias. This was done by dividing the estimates of the source powers by their corresponding estimates of local noise per voxel (Van Veen et al., 1997). By using the Density-Based Spatial Clustering of Applications with Noise (DBSCAN) algorithm (Adelhöfer and Beste, 2020, Ester et al., 1996) as implemented in MATLAB (2020b), the voxels revealed by the DICS beamformer were clustered separately for each frequency band of interest for the contrast trials. All voxels with source activity exceeding two percent of the highest values and within functional neuroanatomical regions based on the Automatic Anatomical Labeling (AAL) atlas (Tzourio-Mazoyer et al., 2002) were selected for clustering. DBSCAN was used to identify neighboring voxels, with an epsilon of once the edge length and a minimum cluster size of two voxels. Contrasts were calculated by subtracting the source power of overlapping Nogo trials from the source power of non-overlapping Nogo trials proportional to the summation of these two conditions. Time windows for the beamforming analysis were based on the time windows of significant classifications in the MVPA for both groups and both frequency bands. Voxels were clustered based on local proximity (Density-Based Spatial Clustering of Applications with Noise (DBSCAN) algorithm) (Ester et al., 1996) and functional neuroanatomical factors (Automatic Anatomical Labeling (AAL) atlas) (Tzourio-Mazoyer et al., 2002). We relied on standard adult brain templates for the study since individual brain scans were not feasible to conduct. Further, to our knowledge, there is no suitable template that captures both children and adolescents of different ages that takes into account the continuously developing brain (Simmonds et al., 2014). Therefore, we utilized a typical adult brain template to get an indication of whether the sources of alpha and theta band activity might significantly differ between adolescents with and without AD(H)D.

### 2.6 Statistical analysis

Behaviorally, statistical differences in conditions were calculated by a repeated measures analysis of variance (RM-ANOVA) including the between-subject factor “group” (AD(H)D vs NT) and the within-subject factor “feature overlap” (non-overlapping vs overlapping) for the performance of Nogo trials (false alarms (FA), i.e., percentage of erroneous reactions). To ensure that those results refer specifically to response inhibition, and not to reacting to conflict in general, another RM-ANOVA with the same factors was conducted for the performance of Go trials (hit rate, i.e., percentage of correct responses, and reaction time (RT)). Further, mean RTs will be reported for successful Go as well as unsuccessful Nogo trials for both groups (AD(H)D, NT) and both conditions (non-overlapping, overlapping). Only correct Nogo trials (i.e., successful inhibition) were analyzed on the neurophysiological level to examine the underlying mechanisms of response inhibition. For all analyses, the difference of non-overlapping minus overlapping conditions denotes the *conflict effect.* The alpha threshold of 0.05 was chosen as significance level. Mean (M) and standard error of the mean (SE) are given for all descriptive statistics and Bonferroni-corrected post-hoc tests are reported when necessary.

Bayesian statistics were used to estimate the strength of the significant and null findings. The Bayes Factor for *BF*_01_ was determined and interpreted by the Bayes Factor classification scheme: a BF value between 1 and 3 as anecdotal, between 3 and 10 as substantial, between 10 and 30 as strong, between 30 and 100 as very strong, and over 100 as extreme evidence for the null hypotheses (H0). On the other hand, the alternative hypothesis (H1) is supported anecdotally by values between 1/3 and 1, substantially by values between 1/10 and 1/3, strongly by values between 1/30 and 1/10, extremely strongly by values between 1/100 and 1/30, and extremely by values less than 1/100. Furthermore, Spearman's correlational analyses will be performed between the subscale ‘hyperactivity/impulsivity’ of the Conners’ Parent Rating Scale and the peak power values of ABA and TBA for Nogo trials of the conflict effect. This will be done to examine whether inhibition related AD(H)D symptoms are correlated to the power modulation of ABA and TBA.

## 3 Results

### 3.1 Behavioral results

**Fig. 2:**
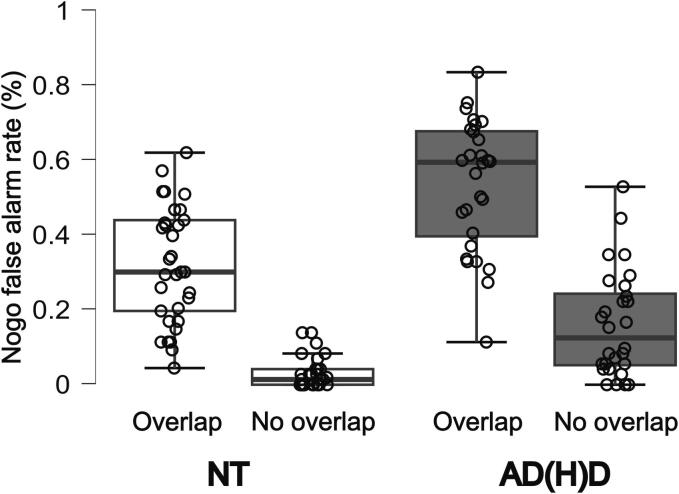
Comparisons of Nogo false alarm rates. The distribution of false alarm rates is shown for both conditions (overlapping, non-overlapping) for the NT group (left side) and the AD(H)D group (right side). Boxes indicate inter-quartile range and median, whiskers extend to values up to 1.5 IQR beyond first and third quartile, respectively. Means of each participant are shown as individual points.

For the FA rate, significant main effects of feature overlap (*F*(1,59) = 390.081, *p* < 0.001, *η_p_^2^* = 0.869, *BF*_01_ < 0.001), group (*F*(1,59) = 30.599, *p <* 0.001, *η_p_^2^* = 0.342, *BF*_01_ < 0.001), as well as a significant interaction between the two (*F*(1,59) = 5.922, *p* = 0.018, *η_p_^2^* = 0.091, *BF*_01_ = 0.420) were revealed. Independent of group, participants showed a significantly higher FA rate in the overlapping NoGo trials (42.8 % ± 2.1 %) compared to the non-overlapping trials (9.5 % ± 1.3 %).This replicates previous findings in healthy adult samples showing similarly high FA rate differences between overlapping and non-overlapping trials (Chmielewski and Beste, 2019, Prochnow et al., 2022b, Prochnow et al., 2022a, Prochnow et al., 2021, Wendiggensen et al., 2023). AD(H)D participants generally showed a significantly higher FA rate in all NoGo trials (34.7 % ± 2.3 %) compared to the NT group (17.6 % ± 2.1 %), which is also in line with previous studies (Hart et al., 2013, Huizenga et al., 2009, Johnstone et al., 2009, Lijffijt et al., 2005, McAuley et al., 2014, Smith et al., 2004). Furthermore, the interaction effect indicates that while AD(H)D participants performed worse in general compared to the NT group, they also seem to be affected more strongly by feature overlap conditions. Specifically, Bonferroni-corrected post-hoc tests indicated that AD(H)D participants compared to NT controls had a significantly higher FA rate in non-overlapping (*M_Diff_* = -0.130, *p* < 0.001, 95 %-CI[-0.182,-0.078]) and in overlapping trials (*M_Diff_* = -0.212, *p* < 0.001, 95 %-CI[-0.297,-0.127]). Moreover, while overlapping compared to non-overlapping trials led to a significantly higher FA rate in the NT group (*M_Diff_* = -0.292, *p* < 0.001, 95 %-CI[-0.337,-0.246]), this led to even higher FA rates in the AD(H)D group (*M_Diff_* = -0.374, *p* < 0.001, 95 %-CI[-0.423,-0.324]). Please refer to Fig. 2 for visualization of the Nogo trials.

**Fig. 3:**
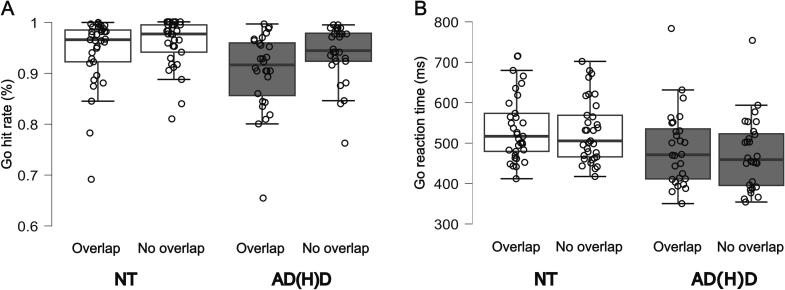
Comparisons of Go hit rate and reaction time. (A) Go hit rate. The distribution of the Go hit rate (percentage of correct responses) is shown for both conditions (overlapping, non-overlapping) for the NT group (left side) and the AD(H)D group (right side). (B) Go reaction time. The distribution of the Go reaction time (in ms) is shown for both conditions (overlapping, non-overlapping) for the NT group (left side) and the AD(H)D group (right side). Boxes indicate inter-quartile range and median, whiskers extend to values up to 1.5 IQR beyond first and third quartile, respectively. Means of each participant are shown as individual points.

To ensure that the interaction of feature overlap and group is specific to response inhibition, accuracy and reaction times (RT) were analyzed for Go trials too. A significant main effect of feature overlap was revealed for accuracy (*F*(1,59) = 25.276, *p* < 0.001, *η_p_^2^* = 0.300, *BF*_01_ < 0.001) and for RT (*F*(1,59) = 14.299, *p* < 0.001, *η_p_^2^* = 0.195, *BF*_01_ = 0.010). Adolescents generally responded more accurately (94.9 % ± 0.7 %) and faster (501.830 ms ± 10.746 ms) in non-overlapping conditions compared to overlapping conditions (92.4 % ± 0.9 %; 511.722 ms ± 11.285 ms). A significant main effect of group was only observed for RT (*F*(1,59) = 5.928, *p* = 0.018, *η_p_^2^* = 0.091, *BF*_01_ = 0.421), but not for accuracy (*F*(1,59) = 3.854, *p* = 0.054, *η_p_^2^* = 0.061, *BF*_01_ = 1.127). AD(H)D participants generally responded faster to a Go trial (480.138 ms ± 16.095 ms) compared to the NT group (533.414 ms ± 14.825 ms). There was no significant interaction effect of feature overlap and group for both accuracy and RT (all *F’s* < 2.802, all *p’s* > 0.099, all *η_p_^2^* < 0.045, *BF*_01_ > 2.191). This underpins the results of the Nogo trials in that participants with AD(H)D are not generally more affected by higher cognitive load (i.e. overlapping conditions) than NT controls, but that this effect may be specific for inhibiting a response. Please refer to Fig. 3 for visualization of the Go trials. For completion, average response speed for successful Go trials as well as unsuccessful Nogo trials are shown in Table 2.

**Table 2:** Average reaction times (mean and SE) of successful Go trials and unsuccessful Nogo trials for both groups (NT, AD(H)D) and both conditions (non-overlapping, overlapping).

	Successful Go trials		Unsuccessful Nogo trials	
	Overlap	Non-overlap	Overlap	Non-overlap
All participants	513.797 ms ± 11.619	504.122 ms ± 11.213	474.149 ms ± 13.959	423.711 ms ± 21.459
NT	537.033 ms ± 14.332	529.796 ms ± 13.678	500.396 ms ± 17.440	500.057 ms ± 38.028
AD(H)D	486.411 ms ± 17.757	473.864 ms ± 16.874	443.215 ms ± 21.285	363.271 ms ± 15.851

Note. In both groups, some participants showed no unsuccessful Nogo trials at all. The means and SE of this condition, are based on N = 19 NT and N = 24 AD(H)D participants.

### 3.2 Neurophysiological Data

#### 3.2.1 Sensor-level activity

**Fig. 4:**
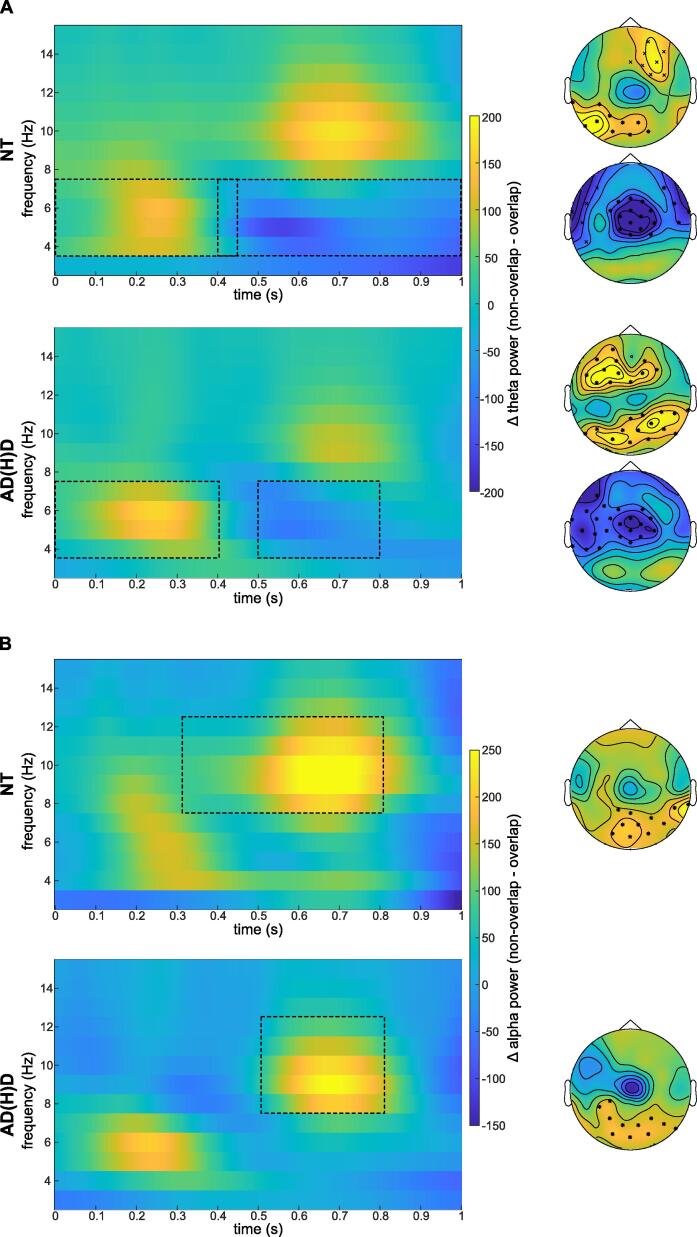
Time-frequency Transformation within groups. Time-frequency transformation and topoplots of significant clusters for the conflict effect (non-overlap minus overlap) within each group. The displayed power is averaged over the electrodes of corresponding clusters within each frequency band. Boxes with dashed lines represent the time windows of corresponding clusters. A) Time-frequency transformation of theta band activity with one positive and one negative clusters per group B) Time-frequency transformation of alpha band activity with one positive cluster per group.

Cluster-based permutation tests indicated significant differences in TBA and ABA for both the NT and AD(H)D group between the non-overlapping and overlapping conditions during the Nogo trials (see Fig. 4).

Specifically, significant positive differences (non-overlapping > overlapping) were revealed for TBA for both groups at parieto-occipital and frontal electrodes, but within the NT group until 450 ms after stimulus onset, (*t_sum_* = 32.99*, p_cluster_* = 0.010 and *t_sum_* = 24.30*, p_cluster_* = 0.018) and within the AD(H)D group until 400 ms after stimulus onset (*t_sum_* = 44.93*, p_cluster_* < 0.001 and *t_sum_* = 40.98*, p_cluster_ <* 0.001). Further, negative differences (non-overlapping < overlapping) in TBA were also revealed: for the NT group at (fronto)central electrodes between 400 – 1000 ms after stimulus onset (*t_sum_* = -70.62*, p_cluster_* = 0.002 and *t_sum_* = –22.77*, p_cluster_* = 0.019), and for the AD(H)D group at frontocentral and frontotemporal electrodes between 500 – 800 ms after stimulus onset (*t_sum_* = -70.72*, p_cluster_* < 0.001). For ABA, cluster-based permutation tests revealed a significant positive difference (non-overlapping > overlapping) for both the NT controls (at parietal-occipital electrodes between 300 to 800 ms after stimulus onset, *t_sum_* = 39.59*, p_cluster_* < 0.001) and the AD(H)D group (at parietal-occipital electrodes between 500 to 800 ms after stimulus onset, *t_sum_* = 26.55*, p_cluster_* = 0.004). Therefore, both groups show similar activity differences: directly after stimulus onset, higher TBA in non-overlapping than in overlapping trials, and later on, lower TBA and higher ABA in non-overlapping than in overlapping trials.

**Fig. 5:**
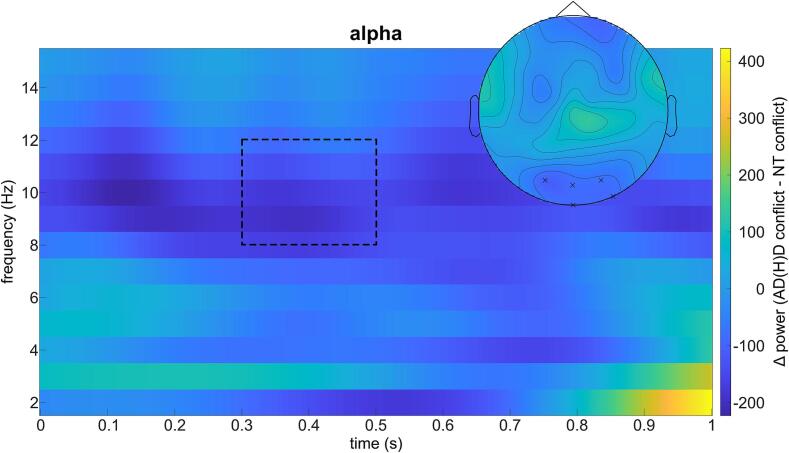
Time-frequency Transformation of group difference. Time-frequency transformation and topoplot of significant cluster for the group difference (ADHD minus NT) of the conflict effect (non-overlap minus overlap). The displayed alpha band power is averaged over the electrodes of the cluster. The box with dashed lines represents the time window of the corresponding cluster.

Comparing the *conflict effect* (non-overlapping – overlapping trials) between the groups, cluster-based permutation tests revealed a significant negative difference (AD(H)D conflict < NT conflict) in ABA at occipital electrodes between 300 to 500 ms after stimulus onset (*t_sum_* = -11.52*, p_cluster_ =* 0.047; Fig. 5). No significant differences were revealed for TBA between the two groups. This suggests that only during the above-mentioned time window, NT controls show a significantly greater difference in ABA between non-overlapping and overlapping trials compared to the AD(H)D group.

#### 3.2.2 Correlational analyses between parent-reported severity of symptoms and neurophysiological data

Spearman’s correlational analyses between the subscale ‘hyperactivity/impulsivity’ of the Conners’ Parent Rating Scale and the peak power values of ABA and TBA for Nogo trials of the conflict effect suggested that the subscale of ‘hyperactivity/impulsivity’ is not significantly correlated to the peak power values of neither ABA, Spearman’s ρ = -0.133, *p* = 0.318, nor TBA, Spearman’s ρ = 0.144, *p* = 0.282.

#### 3.2.3 MVPA on sensor-level TBA and DICS beamforming

**Fig. 6:**
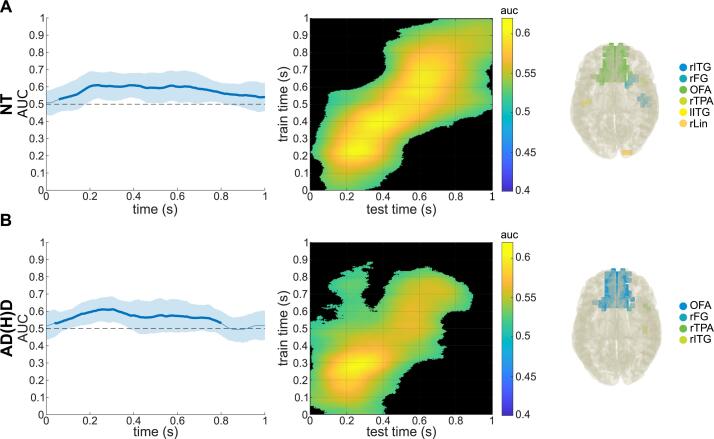
Temporal MVPA results of theta band activity. Temporal MVPA results using theta band training and prediction sets and DBSCAN results of theta band activity in the corresponding time windows within A) the NT sample and B) the ADHD sample. Left plots show the AUC curve over time with the shaded area showing the standard deviation and the thicker line showing when and for how long the classification was accurate significantly over chance-level. Middle plots show the temporal generalization. Right plots depict the clusters of brain regions: A) right inferior temporal gyrus (rITG), right fusiform gyrus (rFG), right and left superior, medial and inferior and right middle orbitofrontal area (OFA), right temporopolar area (rTPA), left inferior temporal gyrus (lITG), and right lingual gyrus (rLin). B) Right and left superior, medial and inferior and right middle orbitofrontal area (OFA), right fusiform gyrus (rFG), right temporopolar area (rTPA), and right inferior temporal gyrus (rITG).

Using theta-band training and prediction sets, the MVPA on the sensor-level theta power time course and subsequent cluster-based permutation testing revealed that the MVPA was able to classify the non-overlapping and overlapping conditions significantly above chance level for both groups (see Fig. 6). For the NT group, the AUC was significant in the time window of approx. 90 to 1000 ms after stimulus onset. For the AD(H)D group, this was the case in the time window of approx. 70 to 770 ms after stimulus onset. Descriptive analyses of the MVPA/AUCs are shown in Table 3. To investigate which brain regions were associated with this, the DBSCAN algorithm was applied for TBA differences (non-overlapping vs overlapping) of each group, and resulting clusters were reallocated based on the AAL atlas. The first cluster in the NT group included right temporal regions (BA 20, B37, B38) and the second cluster included bilateral orbitofrontal and left temporal regions (BA 11, BA 38). For the AD(H)D group, the cluster included bilateral orbitofrontal as well as left temporal regions (BA 11, BA 37, BA 38). Please see Table 4 for a detailed description of all relevant clusters and the corresponding Brodmann areas.

**Table 3:** Results of MVPA on sensor-level theta band activity of the condition classification (non-overlapping vs overlapping).

Group	MVPA across time					Temporal generalization MVPA	
	AUC			Sign. time (ms)	Sign. level	Mean AUC	Pct. sign.
	Mean	Min	Max				
NT	0.581	0.530	0.606	90–––1000	< 0.001	0.559	24.99 %
AD(H)D	0.569	0.527	0.606	70–––770	< 0.001	0.552	16.75 %

Abbreviations: NT = neurotypical, AD(H)D = attention-deficit(−hyperactivity)-disorder, MVPA = multivariate pattern analysis, AUC = area under the curve, sign. time = time interval relative to stimulus onset of significant classification, sign. level) significance level of classification, Pct. Sign. = percentage of classifications with significant AUCs.

**Table 4:** Results of DBSCAN clusters and associated Brodmann areas for averaged theta and alpha band activity of each group (NT, AD(H)D).

Group	Frequency band	Clusters		Figure labels		Neuroanatomical regions
NT	Theta	I		rITG rFGrTPA		Right inferior temporal gyrus (BA 20)Right fusiform gyrus (BA 37)Right superior and middle temporopolar area (BA 38)
		II		OFA lFGlTPA		Right and left superior, medial and inferior and right middle orbitofrontal area (BA 11)Left fusiform gyrus (BA 37)Left superior and middle temporopolar area (BA 38)
	Alpha	I		ITGFG		Right and left inferior temporal gyrus (BA 20)Right and left fusiform gyrus (BA 37)
		II		rITG rPHG FG TPA		Right inferior temporal gyrus (BA 20)Right parahippocampal gyrus (BA 36)Right and left fusiform gyrus (BA 37)Right and left superior and middle temporopolar area (BA 38)
		III		OFA		Right and left superior, medial and inferior and right middle orbitofrontal area (BA 11)
AD(H)D	Theta	I		OFA FGlTPA		Right and left superior, medial and inferior and right middle orbitofrontal area (BA 11)Right and left fusiform gyrus (BA 37)Left superior and middle temporopolar area (BA 38)
	Alpha			OFA FGlTPA		Right and left superior, medial and inferior and right middle orbitofrontal area (BA 11)Right and left fusiform gyrus (BA 37)Left superior and middle temporopolar area (BA 38)

Neuroanatomical regions are based on Brodmann areas and clustered with respect to functional communalities. Abbreviations: NT = neurotypical, AD(H)D = attention-deficit(−hyperactivity)-disorder.

#### 3.2.4 MVPA on sensor-level ABA and DICS beamforming

**Fig. 7:**
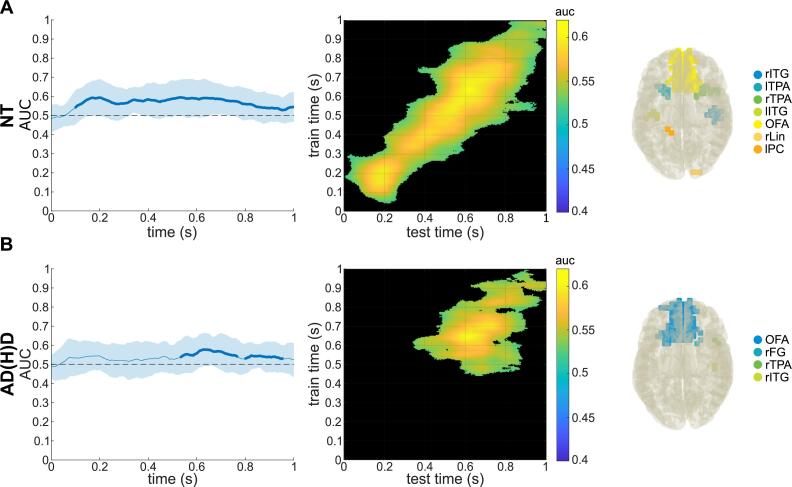
Temporal MVPA results of alpha band activity. Temporal MVPA results using alpha band training and prediction sets and DBSCAN results of alpha band activity in the corresponding time windows within A) the NT sample and B) the ADHD sample. Left plots show the AUC curve over time with the shaded area showing the standard deviation and the thicker line showing when and for how long the classification was accurate significantly over chance-level. Middle plots show the temporal generalization. Right plots depict the clusters of brain regions: A) right inferior temporal gyrus (rITG), left temporopolar area (lTPA), right temporopolar area (rTPA), left inferior temporal gyrus (lITG), left superior, medial and inferior and right middle orbitofrontal area (OFA), right lingual gyrus (rLin), and left postcentral gyrus (lPC). B) Right and left superior, medial and inferior and right middle orbitofrontal area (OFA), right fusiform gyrus (rFG), right temporopolar area (rTPA), and right inferior temporal gyrus (rITG).

Using alpha-band training and prediction sets, the MVPA on the sensor-level alpha power time course and subsequent cluster-based permutation testing revealed that the MVPA was able to differentiate between the non-overlapping and overlapping conditions significantly above chance level for both groups (see Fig. 7). For the NT group, the AUC was significant in the time window of approx. 78 to 1000 ms after stimulus onset, with this being the case in the time window of approx. 520 to 945 ms (with a small ‘break’ between 758 and 805 ms) for the AD(H)D group. Descriptive analyses of the MVPA/AUCs are shown in Table 5. Again, the DBSCAN algorithm was applied for ABA differences (non-overlapping vs overlapping) of each group, and resulting clusters were reallocated based on the AAL atlas. The first (BA 20, BA 37) and second (BA 20, BA 36, BA 37, BA 38) cluster in the NT group included bilateral temporal regions, and the third cluster included bilateral orbitofrontal regions (BA 11). For the AD(H)D group, the significant cluster encompassed also bilateral temporal and bilateral orbitofrontal regions (BA 11, BA 37, BA 38). Again, see Table 4 for a detailed description of all relevant clusters and the corresponding Brodmann areas.

**Table 5:** Results of MVPA on sensor-level alpha band activity of the condition classification (non-overlapping vs overlapping).

Group	Cluster	MVPA across time					Temporal generalization MVPA	
		AUC			Sign. Time (ms)	Sign. level	Mean AUC	Pct. sign.
		Mean	Min	Max				
NT	1	0.566	0.523	0.595	78–––1000	< 0.001	0.551	15.03 %
AD(H)D	1	0.549	0.536	0.564	520–––758	0.002	0.538	7.7 %
	2	0.553	0.529	0.562	805–––945	0.004		

Abbreviations: NT = neurotypical, AD(H)D = attention-deficit(−hyperactivity)-disorder, MVPA = multivariate pattern analysis, AUC = area under the curve, sign. time = time interval relative to stimulus onset of significant classification, sign. level) significance level of classification, Pct. Sign. = percentage of classifications with significant AUCs.

#### 3.2.5 MVPA on sensor-level TBA to ABA prediction

**Fig. 8:**
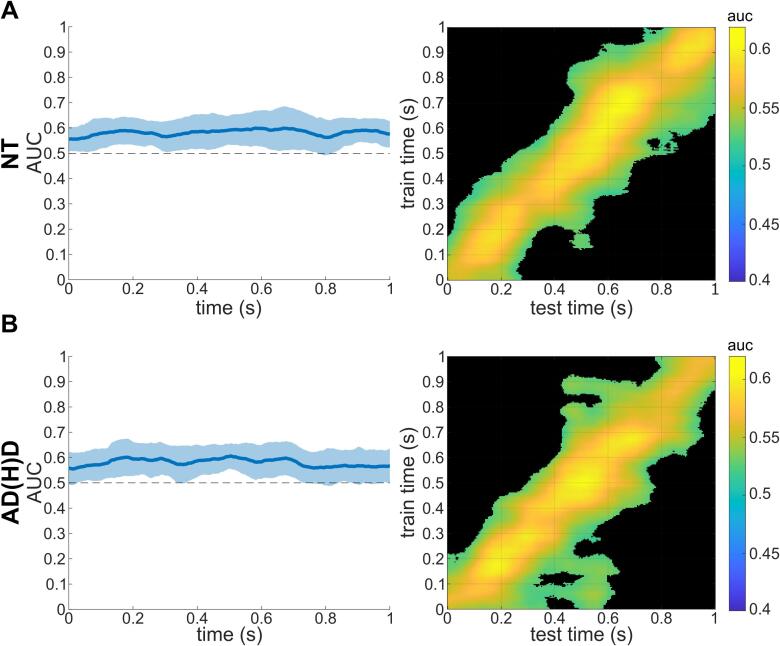
Temporal MVPA results of theta on alpha band activity. Temporal MVPA results using theta band training and alpha band prediction sets within A) the NT sample and B) the ADHD sample. Left plots show the AUC curve over time with the shaded area showing the standard deviation and the thicker line showing when and for how long the classification was accurate significantly over chance-level. Right plots show the temporal generalization.

An MVPA with TBA training and ABA testing was conducted. This was done to explore possible overlapping patterns between both frequency bands (Wendiggensen et al., 2023). The MVPA on the sensor-level and subsequent cluster-based permutation testing revealed that the MVPA was able to differentiate between the non-overlapping and overlapping conditions significantly above chance level for both groups across the whole trial length (see Fig. 8). Descriptive analyses of the MVPA/AUCs are shown in Table 6.

**Table 6:** Results of MVPA on sensor-level theta band on alpha band activity of the condition classification (non-overlapping vs overlapping).

Group	MVPA across time					Temporal generalization MVPA	
	AUC			Sign. Time (ms)	Sign. level	Mean AUC	Pct. sign.
	Mean	Min	Max				
NT	0.582	0.556	0.600	0–––1000	< 0.001	0.556	46.06 %
AD(H)D	0.581	0.555	0.606	0–––1000	< 0.001	0.555	46.97 %

Abbreviations: NT = neurotypical, AD(H)D = attention-deficit(−hyperactivity)-disorder, MVPA = multivariate pattern analysis, AUC = area under the curve, sign. time = time interval relative to stimulus onset of significant classification, sign. level) significance level of classification, Pct. Sign. = percentage of classifications with significant AUCs.

## 4 Discussion

Though deficits in inhibitory control are a main symptom of AD(H)D, leading to serious everyday issues, a detailed understanding of why and under which circumstances these deficits occur is missing. The objective of the present study was to examine if and how inhibitory control of adolescents with AD(H)D is more affected by increased cognitive load compared to NT adolescents, and what underlying neurophysiological mechanisms are associated with this. Performance of the AD(H)D participants was impaired compared to NT controls, especially in conditions with higher cognitive conflict (i.e., overlapping trials). Neurophysiological data suggests that this is not due to a generally impaired activation of relevant brain regions or a failed modulation of TBA, but rather due to the inefficiency in modulating ABA in time.

In line with earlier research (Chmielewski and Beste, 2019, Prochnow et al., 2024, Prochnow et al., 2022b, Prochnow et al., 2022a, Prochnow et al., 2021, Wendiggensen et al., 2023), the behavioral results replicate findings showing that inhibition is generally less successful in higher compared to lower cognitive demand conditions. This was indicated by the higher false alarm rate (i.e., percentage of errors) in the overlapping compared to the non-overlapping trials. Previous research suggested that dealing with conflicts increases the need to update existing internal representations, leading to the observed impairments in performance (Prochnow et al., 2022b, 2022a, 2021). Further, consistent with past research (Hart et al., 2013, Huizenga et al., 2009, Johnstone et al., 2009, Lijffijt et al., 2005, Lipszyc and Schachar, 2010, McAuley et al., 2014, Smith et al., 2004), the current study suggests that adolescents with AD(H)D are less successful in inhibiting responses and, more importantly, show a higher discrepancy in their inhibitory control between low and high conflict conditions. Behaviorally speaking, this was indicated by the generally significantly heightened false alarm rate in AD(H)D participants compared to the NT controls independent of the stimulus feature overlap between Go and Nogo trials. Moreover, AD(H)D participants’ performance was specifically impaired (heightened false alarm rate) when they were additionally confronted with high conflict conditions (overlapping trials).

By that, the study reveals that the AD(H)D sample at hand indeed showed deficient response inhibition processes compared to NT controls in line with earlier research on response inhibition deficits in AD(H)D employing a GoNogo paradigm (e.g., Chmielewski et al., 2019, Hoegl et al., 2012, Johnstone et al., 2009, Johnstone and Clarke, 2009, Smith et al., 2004, Wright et al., 2015). More to that, their performance becomes even more compromised when a cognitive conflict is superimposed. This suggests cognitive conflict to be one influencing factor in enhancing response inhibition deficits, specifically when those deficits were already established before. Considering that impulsivity is one of the main characteristics of adolescent AD(H)D (e.g., Barkley, 1997, Nigg, 2001) and that the disorder is linked to a tendency toward automatic behavior (Clark et al., 2000), it is possible that the high degree of automaticity in response tendencies further compromised the performance in the applied GoNogo paradigm. Generally, despite a study investigating the influence of multimodal conflicts on inhibitory control in AD(H)D (Chmielewski et al., 2018), the impact of conflict-modulating factors on response inhibition processes in AD(H)D have rarely been studied in detail. The study reveals as such new insights into the factors modulating response inhibition processes in patients with AD(H)D, being one of the first combining the heightened demand to inhibit a response while at the same time updating existing mental representations.

Earlier research suggested that a specific interplay of upregulating TBA and downregulating ABA plays a crucial role in successful inhibitory control, especially when confronted with changes in cognitive demands (Wendiggensen et al., 2023). Can the observed behavioral group differences thus be attributed to deficient up- or downregulation of the necessary frequency bands? This assumption is only partly supported. Just like it had been shown before (Prochnow et al., 2022a, Prochnow et al., 2021, Wendiggensen et al., 2023), our findings demonstrate that a conflict-dependent modulation of ABA and TBA was present in all participants. In cognitively more demanding trials, both groups showed significantly higher TBA and lower ABA, whereas in the cognitively less demanding trials, the opposite was true. This is in line with work suggesting that frontal TBA increases in response to conflict since an increased monitoring of cognitive processes and an update of mental representations are necessary (Prochnow et al., 2022a, 2021). Further, stronger parietal-occipital ABA modulations supposedly reflect a more efficient information processing when cognitive demand is low, thereby facilitating the inhibition of task-irrelevant processes (e.g., Janssens et al., 2018).

Yet, the current study also suggests differences to previous work: First, these younger participants showed significantly more parieto-occipital and frontal TBA in low-conflict trials just after appearance of the stimuli. This activity changed to more fronto(central) and for the AD(H)D group also to additional frontotemporal TBA during high-conflict trials around the time of the response for both groups. An explanation could be that in the low-conflict trials, the retrieval process (which has been linked to increased TBA; Beste et al., 2023, Wendiggensen et al., 2023) starts right away since the stimulus is unambiguous, but is delayed in high-conflict (i.e., ambiguous) trials. Moreover, while AD(H)D participants revealed a similar modulation pattern as the NT controls, they seemed to be less efficient at processing the relevant information on time. More specifically, AD(H)D participants seem to modulate ABA around 200 ms later than NT controls. This means that they appear to start differentiating between both conflict conditions later, which results in the condition-appropriate regulation of ABA to be delayed. This finding is substantiated by the results of the between-groups analysis of the *conflict effect* (non-overlapping − overlapping). The AD(H)D group showed a significantly smaller occipital ABA difference between the two conditions compared to the NT controls, precisely in the time window in which the NT group had already demonstrated the necessary significant ABA modulation. Moreover, when taking the reaction time of unsuccessfully inhibited Nogo trials (i.e., false alarms) into account, this delayed differentiation in AD(H)D participants took place around the time when they, on average, already failed to suppress the corresponding response. Thus, our study demonstrates that, in AD(H)D, the necessary ABA modulation to dissolve the higher cognitive demand commences often only after the required motor output.

This occipital ABA modulation has previously been linked to retrieval processes of pre-existing mental representations, enhanced attentional control for filtering of irrelevant information as well as proposed to function as a top-down control over TBA (Beste et al., 2023, Janssens et al., 2018, Prochnow et al., 2022a). This is further corroborated by the MVPA results: while the classifier on TBA-basis could differentiate the non-overlapping and overlapping condition for both groups almost across the whole trial, the classifier on ABA-basis showed a different pattern between the groups. More specifically, while the MVPA was able to distinguish between the two conditions and preserve the representational differences for nearly the entire trial duration in the NT group, this was only the case for approximately half of the trial length in the AD(H)D group. Importantly, consistent with prior research (Wendiggensen et al., 2023), upon examining the MVPA classification between both frequency bands, it was revealed that representational differences of TBA can also be applied to ABA to reliably distinguish the two conditions, further suggesting the importance of their interplay for successful response inhibition with heightened cognitive load.

Interestingly, by assessing the sources underlying TBA and ABA for the contrast between both conditions, it was shown that similar brain areas were activated in both groups (i.e., temporal and orbitofrontal regions). This is consistent with earlier research showing that specifically the inferior temporal gyrus, the temporal pole, and the fusiform gyrus seem to be involved in the categorization and identification of visual stimuli and as such play a role in retrieving existing mental representations in the task at hand (Dehaene and Le Clec’H, G., Poline, J.-B., Le Bihan, D., Cohen, L., , 2002, Devlin et al., 2006, Forseth et al., 2018, Goodale et al., 2005, Grill-Spector and Weiner, 2014, Haxby et al., 2001). Further, the importance of temporal regions for retrieval of these representations is supported by their role in episodic memory retrieval (Baddeley et al., 2001, Kwok et al., 2012, Nyberg et al., 1996, Wiggs et al., 1998). Orbitofrontal areas, on the other hand, likely receive memory information necessary for retrieval of representations (Eggert et al., 2022), are linked to the inhibitory control network (Bari and Robbins, 2013, Horn et al., 2003), and also seem to play a role in the adaptation of goal-directed behavior (Rolls, 2004, Rudebeck and Rich, 2018).

Overall, behavioral and neurophysiological evidence suggests that adolescents with AD(H)D seem to be more strongly affected by increased response inhibition demands through additional conflicts. Further, findings indicate that behavioral deficits in AD(H)D are likely based on a delayed top-down modulation of ABA. This in turn, leads to belated monitoring of cognitive processes and updating of mental representations of working memory content. This pattern of results does not seem to stem from deficiencies in TBA modulation per se or from differences in activated brain structures.

A limitation of the study is the interindividual variability of the adolescent sample at hand. Brain regions are likely still undergoing considerable changes until early adulthood (Dennis and Thompson, 2013, Rubia, 2013, Stiles and Jernigan, 2010). Thus, while both behavioral and neurophysiological results of the study mainly replicated findings of earlier research regarding NT adults, it would be of interest for future studies to take a developmental perspective on response inhibition processes under conflict and consider using individual brain scans. Recent literature suggests that gender differences in cognitive development appear to be rather small and may only have a minimal influence on the results on different cognitive tasks (Grissom and Reyes, 2019, Hyde, 2016, Hyde, 2005, Hyde et al., 2019). Nevertheless, we cannot rule out for sure that gender did not play a role in the observed effects. Hence, future studies would benefit from matching the two groups by gender, and by that, controlling for balanced gender ratios in the sample. Furthermore, AD(H)D symptoms, specifically the severity of impulsivity and response inhibition deficits, can vary widely between patients, suggesting that a generalization to the broader patient population needs to be done cautiously.

This study is one of the first to investigate inhibitory control deficits under high cognitive load due to pre-existing mental representations in need of updating. Findings suggest that in addition to their general deficits in response inhibition, adolescents with AD(H)D show specific deficiencies when confronted with increased response inhibition demands by additional conflict. Most importantly, findings show that this seems mainly due to a delay of ABA modulation and not due to TBA abnormalities, differences in activated brain regions, or a change in the TBA-ABA-interplay. In summary, the present study provides novel insights into underlying neurophysiological mechanisms of the complex nature of response inhibition deficits in adolescents with AD(H)D.

## 5 Funding sources

This work was supported by the Federal Ministry of Education and Research (Bundesministerium für Bildung und Forschung, BMBF) as part of the German Center for Child and Adolescent Health (DZKJ) under the funding code 01GL2405B to Christian Beste. The funder played no role in the decision to submit the paper or the presentation of the content.

## 6 Disclosures

All authors report no biomedical financial interests or potential conflicts of interest.

## CRediT authorship contribution statement

**Katharina Graf:** Writing – review & editing, Writing – original draft, Visualization, Investigation, Formal analysis, Conceptualization. **Roula Jamous:** Writing – review & editing, Writing – original draft, Visualization, Software, Investigation, Formal analysis, Conceptualization. **Moritz Mückschel:** Writing – review & editing, Software, Methodology, Conceptualization. **Annet Bluschke:** Writing – review & editing, Writing – original draft, Supervision, Funding acquisition, Conceptualization. **Christian Beste:** Writing – review & editing, Writing – original draft, Validation, Supervision, Resources, Project administration, Methodology, Funding acquisition, Conceptualization.

## Declaration of competing interest

The authors declare that they have no known competing financial interests or personal relationships that could have appeared to influence the work reported in this paper.

## Data Availability

THe link to the OSF repository where all data has been stored is included in the manuscript.
